# Complete genome sequence of *Bacillus halotolerans* F29-3, a fengycin-producing strain

**DOI:** 10.1128/mra.01246-23

**Published:** 2024-03-07

**Authors:** Hung-Yu Shu, Chien-Chi Chen, Hsin-Tzu Ku, Chun-Lin Wang, Keh-Ming Wu, Hui-Ying Weng, Shih-Tung Liu, Chyi-Liang Chen, Cheng-Hsun Chiu

**Affiliations:** 1 Department of Bioscience Technology, Chang Jung Christian University, Tainan, Taiwan; 2 Bioresource Collection and Research Center, Food Industry Research and Development Institute, Hsinchu, Taiwan; 3 Bioinformatics Department, Welgene Biotech Co., Ltd., Taipei, Taiwan; 4 Biomedical Industry Ph.D. Program, National Yang Ming Chiao Tung University, Taipei, Taiwan; 5 Department of Microbiology and Immunology, College of Medicine, Chang Gung University, Taoyuan, Taiwan; 6 Molecular Infectious Disease Research Center, Chang Gung Memorial Hospital, Taoyuan, Taiwan; 7 Division of Pediatric Infectious Diseases, Department of Pediatrics, Chang Gung Memorial Hospital, Taoyuan, Taiwan; University of Maryland School of Medicine, Baltimore, Maryland, USA

**Keywords:** *Bacillus halotolerans*, *Bacillus subtilis*, fengycin, non-ribosomal peptide synthetase, polyketide synthase

## Abstract

*Bacillus halotolerans* F29-3, a Gram-positive bacterium, is recognized for its synthesis of the antifungal substance fengycin. This announcement introduces the complete genome sequence and provides insights into the genetic products related to antibiotic secondary metabolites, including non-ribosomal peptide synthetase (NRPS), polyketide synthase (PKS), and NRPS/PKS combination.

## ANNOUNCEMENT

**Fig 1:**
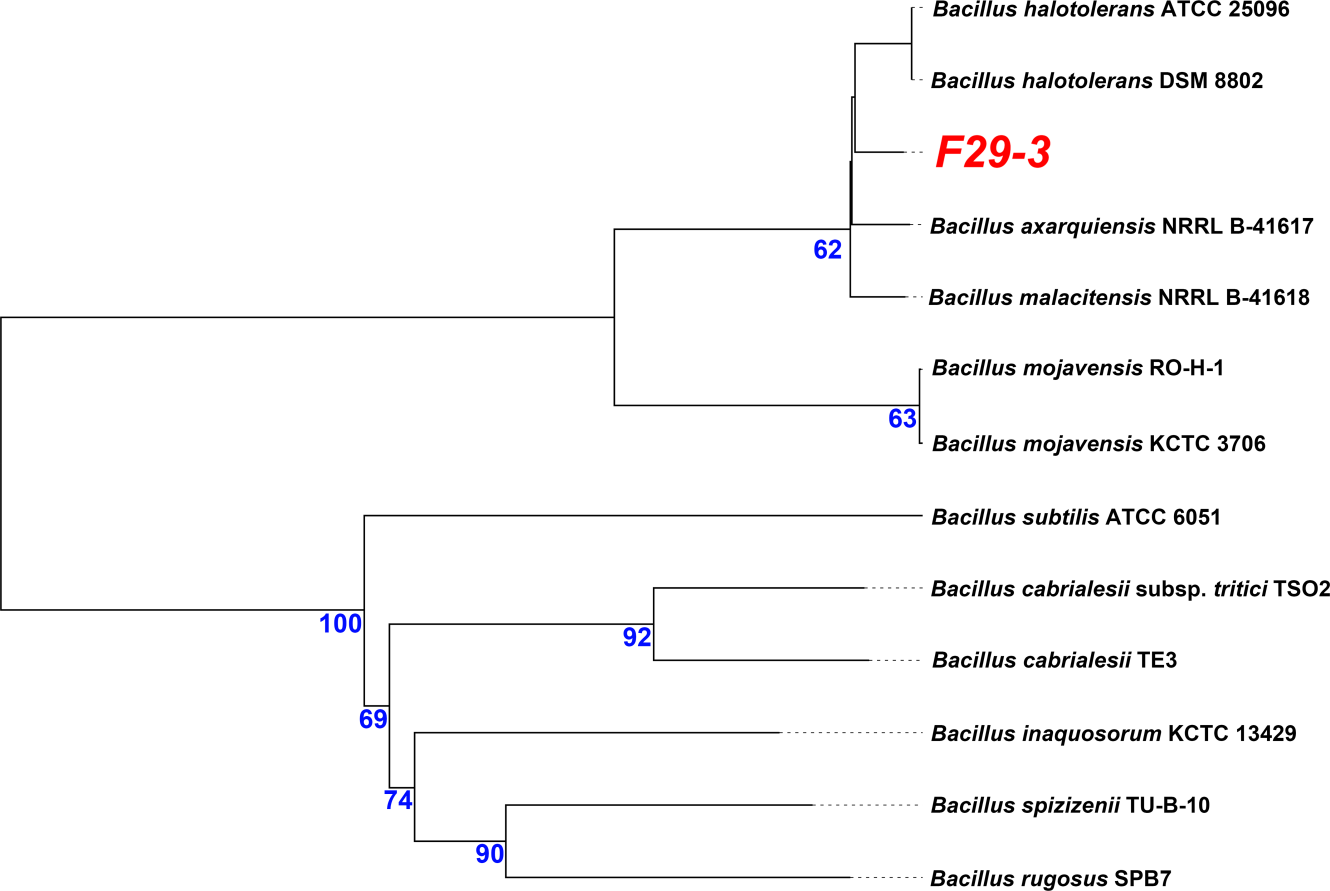
A phylogenetic tree of the F29-3 strain’s genome was generated using the TYGS (accessed on 26 November 2023). The lengths of the branches in the tree are proportional to the Genome BLAST Distance Phylogeny (GBDP) distance formula d5. The numerical values displayed along the branches represent the GBDP pseudobootstrap support values, all exceeding 60% out of 100 replications, with an average branch support of 85.8%.

The *Bacillus halotolerans* strain F29-3 (Fig. 1), previously categorized as *Bacillus subtilis*, was reclassified within the *Bacillus mojavensis* subgroup and *B. subtilis* group using the Type (Strain) Genome Server (TYGS) platform (1). This strain was discovered in a potato cultivation region affected by an outbreak caused by pathogenic filamentous fungus *Rhizoctonia solani*. F29-3 is recognized for its ability to produce fengycin, an antifungal compound named after its place of origin (Fengyuan District, Taichung, Taiwan). The genetic and functional validation of the *fenCDEAB* loci, encoding five non-ribosomal peptide synthetases (NRPSs) crucial for fengycin synthesis, was conducted (2–8).

Numerous *Bacillus* species have been found to have commercial applications in safeguarding and promoting plant root growth due to their features to combat bacteria and fungi, as well as human cancer cells, through the production of secondary metabolites. These include cyclic lipopeptides like bacillibactin, bacillomycin-D, fengycin, and surfactin, as well as polyketides like bacillaene, difficidin, and macrolactin (9–14).

The F29-3 strain was originally isolated in 1977 in a yeast extract–malt extract–D-glucose agar medium overnight at 37°C (15). The genomic DNA of the strain cultured in Luria-Bertani broth medium at 37°C for 4-hour subculture was extracted using NucleoBond Buffer Set III and NucleoBond AXG 20 columns (MACHEREY-NAGEL, Germany) and sheared into 10‒15 Kb fragments (*g*-TUBE, Covaris, USA), underwent purification (AMPure PB beads, Pacific Biosciences, USA) and quantification (Femto Pulse System, Agilent, USA) before library preparation (SMRTbell Express Template Prep Kit 2.0, Pacific Biosciences, USA) with subsequent size selection (BluePippin, Sage Science, USA). The library, sequenced with 779-fold coverage on the PacBio Sequel IIe platform using PacBio 2.0 chemistry, produced 45,208 polymerase reads, amounting to 3,548,728,981 bp, among which were 367,118 subreads with an *N*_50_ value of 10,921 bp. Genome assembly using the Hierarchical Genome Assembly Process version 3.0 pipeline with default parameters integrated into the PacBio SMRT Link software (PacBio, version 10.1.0.119588) (16) yielded a singular assembled chromosome. Manual circularization was performed to remove overlapping ends. Annotation was executed using the DDBJ Fast Annotation and Submission Tool (version v.1.5.0) (17). Unless specified otherwise, default parameters were applied across all software utilized.

The genome spans a length of 4,195,508 bp, exhibiting a 99.30% similarity to that of *B. halotolerans* ATCC 25096. It unveils a total of 4,203 gene sequences, including 4,086 protein-coding genes, 30 rRNA genes (comprising 5S, 16S, and 23S), and 87 tRNA genes.

Using the antiSMASH 6.0 pipeline (18, 19), genome mining for antibiotics and secondary metabolite assessment revealed a diverse array of biosynthetic gene clusters. Among these, a notable focus was on those responsible for encoding NRPS and polyketide synthases (PKSs), which are implicated in potential antibacterial, antifungal, and antitumor properties. The predicted compounds generated by NRPSs, PKSs, and NRPS/PKS combinations include surfactin (encoded by *srf*), novel polyketide (*hal*), bacillaene (*bae*), iturin (*itu*), fengycin (*fen*), isoprenylcysteine carboxyl methyltransferase (a type III polyketide synthase, *BSF_21240*), bacillibactin (*dhb*), and bacilysin (*bac*) (15, 18–30). Furthermore, certain other substances (which are not products of NRPSs or PKSs) also possess potential antibacterial properties, including putative terpene (*sod*), subtilosin (a macrocyclic bacteriocin, *alb*), and epipeptide (*lia*) (Table 1).

**TABLE 1:** List of biosynthetic gene clusters that are involved in the synthesis of antibiotics and secondary metabolites, particularly related to NRPS/PKS, in the genome of strain F29-3

Secondary metabolites (locus_tag)*^a^*	Locus	Antibiotics and secondary metabolite (location)	Activities (citation)	Size (kb)
1. Surfactin (BSF_03510‒BSF_03540; BSF_03590)	*Srf* (*sfp*)	NRPS: *tlpC_srfAA-srfAC_YcxB:* (374,639–406,870)	Putative antibacterial and antifungal properties; anti-mycoplasma, antiviral, anti-inflammatory, and thrombolytic activities (20)	32.2
2. Novel polyketide (BSF_06950‒BSF_07070)	*hal* (*pks*)	PKS: PKS-like_T1PKS: (738,846–746,238) transAT-PKS: (749,436–806,935) T3PKS: (807,115–808,380)	Putative antibacterial, antifungal, anticancer, antiviral, immune-suppressing, anti-cholesterol, and anti-inflammatory activities (21)	69.5
3. Bacillaene (89,319 nt) (BSF_17520‒BSF_17790)	*bae* (*pks*)	Hybrid NRPS-PKS-NRPS: transAT-PKS: (1,821,575–1,910,893) PKS-like: (1,821,575–1,842,067) T3PKS: (1,842,068–1,843,330) alpha-hydroxy-isocaproic-acid: (1,844,956–1,860,108) NRPS: (1,844,956–1,860,108) NRPS: (1,886,715–1,903,202) transAT-PKS-like: (1,886,715–1,910,893)	Putative antibacterial (22, 23)	89.3
4. A. Iturin (BSF_19090‒BSF_19270) B. Fengycin (BSF_19120‒BSF_19320)	A. *itu* B. *fen*	A. Hybrid PKS-NRPS: *fabD*: (2,053,304–2,054,506) transAT-PKS/NRPS: (2,017,303–2,054,506) B. Hybrid PKS-NRPS: transAT-PKS (betalactone): (2,041,339–2,073,095) *fenC‒fenB*: (2,069,271–2,107,054)	A. Lipopeptide: putative insecticidal, antibacterial, hemolytic, and anticarcinogenic properties (24) B. Lipopeptide: putative antifungal, antitumor, and antiadhesive effects (25)	A. 51.8 B. 65.7
5. Terpene (BSF_20290)	*sod*	*sodF:* (2,205,519–2,208,252)	Putative terpenoid: antibacterial; aromatic flavor, such as beta carotene (26)	2.7
6. Isoprenylcysteine carboxyl methyltransferase (ICMT) (BSF_21240)	*caa*	PKS: T3PKS: (2,287,737–2,289,344)	Predicted integral membrane enzyme: substrate recognition and signal transduction; putative ICMT inhibitor: induces cancer cell death and attenuates tumor growth (27)	1.6
7. Bacillibactin (BSF_31300‒BSF_31360)	*dhb*	NRPS: *dhbF–dhbA*: (3,232,785–3,244,559) ferri-bacillibactin esterase BesA: (3,244,747–3,245,610)	Putative NAD + oxidoreductase: participate in the biosynthesis of siderophore group nonribosomal (28)	12.8
8. Sactipeptide: subtilosin (BSF_36970‒BSF_37040)	*alb*	*sboA_albA-albG*: (3,803,230–3,810,169)	Putative subtilosin (macrocyclic bacteriocin): antibacterial action of subtilosin against *Gardnerella vaginalis* (29)	6.9
9. Bacilysin (BSF_37290‒BSF_37350)	*bac*	NRPS: *bacA-bacG*: (3,833,577–3,840,277)	Bacilysin: antibacterial dipeptide (15, 21)	6.7
10. Epipeptide (BSF_39820‒BSF_39830)	*lia*	*YydF-liaK*: (4,095,408–4,097,109)	Predicted epipeptide: a competition determinant (30)	1.7

^*a*^ AntiSMASH version 6.0—a comprehensive resource for the genome mining of biosynthetic gene clusters (18, 19).

## Data Availability

The complete genome sequence of strain F29-3 is available in DDBJ/EMBL/GenBank under accession no. AP014694 (BioProject: PRJDB3291; BioSample: SAMD00023967). The raw data have been deposited in the SRA under the accession number DRA017558.
